# Electrospun Produced 3D Matrices for Covering of Vascular Stents: Paclitaxel Release Depending on Fiber Structure and Composition of the External Environment

**DOI:** 10.3390/ma11112176

**Published:** 2018-11-02

**Authors:** Konstantin A. Kuznetsov, Alena O. Stepanova, Ren I. Kvon, Timothy E. L. Douglas, Nikita A. Kuznetsov, Vera S. Chernonosova, Ivan A. Zaporozhchenko, Maria V. Kharkova, Irina V. Romanova, Andrey A. Karpenko, Pavel P. Laktionov

**Affiliations:** 1 Institute of Chemical Biology and Fundamental Medicine, Siberian Branch, Russian Academy of Sciences, Novosibirsk 630090, Russia; lebedeva@niboch.nsc.ru (A.O.S.); Nikita.Kuznetsov@niboch.nsc.ru (N.A.K.); vera_mal@niboch.nsc.ru (V.S.C.); ivanzap@niboch.nsc.ru (I.A.Z.); kharkova@niboch.nsc.ru (M.V.K.); irin-romanova@yandex.ru (I.V.R.); lakt@niboch.nsc.ru (P.P.L.); 2 Meshalkin National Medical Research Center, Ministry of Health of the Russian Federation, Novosibirsk 630055, Russia; andreikarpenko@rambler.ru; 3 Boreskov Institute of Catalysis, Siberian Branch, Russian Academy of Sciences, Novosibirsk 630090, Russia; kvon@catalysis.ru; 4 Engineering Department, Lancaster University, Lancaster LA1 4YW, UK; t.douglas@lancaster.ac.uk; 5 Materials Science Institute (MSI), Lancaster University, Lancaster LA1 4YW, UK

**Keywords:** drug release, electrospinning, paclitaxel, polycaprolactone, 3D matrix

## Abstract

Paclitaxel is a natural, highly lipophilic anti proliferative drug widely used in medicine. We have studied the release of tritium-labeled paclitaxel (^3^H-PTX) from matrices destined for the coating of vascular stents and produced by the electrospinning method from the solutions of polycaprolactone (PCL) with paclitaxel (PTX) in hexafluoisopropanol (HFIP) and/or solutions of PCL with PTX and human serum albumin (HSA) in HFIP or HIFP-dimethyl sulphoxide (DMSO) blend. The release of PTX has been shown to depend on the composition of electrospinning solution, as well as the surrounding medium, particularly the concentration of free PTX and PTX-binding biomolecules present in human serum. It was shown that 3D matrices can completely release PTX without weight loss. Two-phase PTX release from optimized 3D matrices was obtained: ~27% of PTX was released in the first day, another 8% were released over the next 26 days. Wherein ~2.8%, ~2.3%, and ~0.25% of PTX was released on day 3, 9, and 27, respectively. Considering PTX toxicity, the rate of its diffusion through the arterial wall, and the data obtained the minimum cytostatic dose of the drug in the arterial wall will be maintained for at least three months.

## 1. Introduction

Nano- and microfiber-assisted drug delivery is actively studied worldwide. It is used in specific areas, such as tissue engineering [1], but also to solve general problems of drug delivery, such as enhancement of drug solubility [2] and tailoring of the kinetics of drug release [3]. Electrospinning (ES), i.e., the formation of polymer filaments from melts or solutions of polymers in a strong electric field, is a suitable method for the incorporation of drugs into the fibers and is frequently used for production of drug-enriched matrices. Nano- and microfibers produced by ES have a large surface area. Furthermore, their porosity, hydrophilicity/hydrophobicity, ability participate in to ionic and nonionic interactions, as well as diameter and ultrastructure of the fibers can be tailored. In addition, it is possible to use polymers possessing different degradation rates [4]. The most convenient is ES of polymer solutions, which can employ one or more polymers and drugs, emulsions, and suspensions of drugs [5]. Fiber surface and porosity are usually modified by adding water-soluble biopolymers or low-molecular-weight components to the ES solution or using water- or thermo-induced phase separation [6]. The fibers of different structure can be produced by ES, including hollow, coaxial, and (multi)double-layered fibers combined with drugs introduced in different manners. These fibers can be produced simultaneously forming multi-fiber matrices or several different types of fibers, which can be arranged layer-by-layer in order to form multilayered matrices with different properties assigned to each layer [7]. The benefits of this approach are readily acknowledged, and electrospinning is widely used to fabricate drug-loaded materials, including bactericidal antibiotic dressings [8], growth factor-loaded 3D matrices capable of inducing cell proliferation and wound healing [9], and matrices with cytostatic drugs, used as anti-adhesive membranes [10]. Moreover, ES can used to arrange production lines that combine synthesis of active agents with co-synthetic formulation [11], which can potentially be applied to easily producible drug-loaded matrices.

Recently, electrospun matrices were suggested as coatings for bare-metal esophageal [12] and vascular stents [13]. The high incidence of restenosis requires the reassessment and improvement of current practices for the remodeling of stented arterial regions with bare-metal stents [14]. Restenosis is caused by the proliferation of smooth muscle cells of the arterial wall, endothelial cells, or cells of atherosclerotic plaques induced by the mechanical action on the remodeled vessel, damage of the endothelial layer, and other surrounding cell layers/tissues [15]. Induction of local inflammation in the stent region also promotes cell proliferation and neointima growth [16]. Drug-eluting stents covered with anti-proliferative or anti-inflammatory drugs, such as sirolimus and paclitaxel (PTX), were proposed to reduce the proliferation of surrounding cells. PTX is a natural, highly lipophilic, water-insoluble compound, which exhibits a cytotoxic antimitotic effect by activating the assembly of microtubules from tubulin dimers, stabilizing microtubules, and inhibiting the reorganization of the microtubular network in the interphase and during mitosis [14]. The lipophilicity of PTX allows its accumulation in the altered atheromatous vascular wall. The usual dose of PTX in paclitaxel-eluting stents is approximately 3 µg/mm^2^ [17]. Excessive doses were shown to induce the formation of aneurysms [18]. Therefore, blending of PTX with polymers was proposed to increase the effect of PTX i.e., short-term and long-term toxicity, while using lower concentrations of PTX. For example, the Eluvia stents (Boston Scientific Corporation, PTX with poly(vinylidene fluorideco-hexafluoropropylene) only contain 0.167 µg/mm^2^ PTX [19].

In this work, we studied the release of PTX from the 3D matrices prepared from a solution of polycaprolactone (PCL) containing human serum albumin (HSA) intended for the coating of bare-metal stents. Such matrices have good mechanical characteristics and an extended region of elastic deformation, i.e., if used as a coating, they will not exert additional loads on struts after stent expansion during installation. Moreover, the slow degradation rate of PCL may allow it to provide a lasting mechanical protection of the vessel lumen from stenosis. It is implemented in a similar way in the Inspire MD CGuard™ stents [20]. Release of PTX from matrices can be slowed by adding HSA, which binds PTX at K_d_ = 1.43 × 10^4^ M^−1^ [21] and acts as the main carrier of PTX in the human body. In addition, HSA has been shown to reduce platelet adhesion and increase thromboresistance and hemocompatibility of blood-exposed surfaces [22,23]. With the task of creating a coating that can prevent cell proliferation for as long as possible, we developed and fabricated 3D matrices enabling prolonged PTX delivery. 3D matrices were produced by electrospinning and characterized by general methods, including tensile strength, SEM, XPS, contact angle, etc., PTX release was studied using tritium-labeled PTX.

## 2. Materials and Methods

### 2.1. Production and Quality Control of Tritium-Labeled Paclitaxel

Tritium-labeled PTX (^3^H-PTX) was synthesized by thermoactivated tritium exchange as described earlier [24]. ^3^H-PTX was purified from by-products by reverse phase chromatography on a C18 column using a gradient of acetonitrile in water (25–100%). The radiochemical purity of the resulting compound was evaluated by autoradiography after thin layer chromatography (TLC) on Kieselgel 60 F254 plates (Merck, Germany, 25 Alufolien 20 cm × 20 cm) in a chloroform-methanol-water mixture (19:1:0.1, Rf~0.7). Radioactivity of the preparation was measured on a Tri-Carb 2800 TR β-counter (PerkinElmer, Waltham, MA, USA) in a “ULTIMA GOLD LTT” scintillator (Perkin Elmer, Waltham, MA, USA). An aliquot of the sample (0.1 mL) was thoroughly mixed with a scintillator (0.9 mL), and radioactivity was measured at the same time after preparation of the mixture.

### 2.2. Preparation of 3D Matrices by Electrospinning

Electrospinning solutions were prepared using stock solutions of 9% PCL and 1% HSA (Sigma-Aldrich, St. Louis, MI, USA) in 1,1,1,3,3,3-hexafluoroisopropanol (HFIP, Sigma-Aldrich, USA). The HSA concentration in matrices is given as weight percentage (wt/wt) of total matrix weight. PTX (Sigma-Aldrich, USA) was dissolved in HFIP or DMSO (Sigma-Aldrich, USA), and added to the matrix (~0.46 μg/cm^2^, which corresponds to 0.36 μg/disk). DMSO (3 or 6%, *v*/*v*) was added to the solution of polymers. ^3^H-PTX was diluted with unlabeled PTX to provide at least 26,000 cpm/cm^2^ (or 20,000 cpm/disk, 10 mm diameter disk, ~0.785 cm^2^). To produce the 3D matrices with ^3^H-PTX, a homemade electrospinning device with an airproof chamber and exhaust HEPA filter was used, equipped with a Spellman SL 150 (30 kV, Spellman, Brockton, MA, USA) power supply. Matrices of a thickness of 150–180 µm were prepared using a drum collector 2 cm in diameter and 5.2 cm in length (32.6 cm^2^) under the following conditions: Feed rate, 1.2–1.4 mL/h; capillary-collector distance, 19–20 cm; voltage, 23–25 kV; collector rotation speed, 300 rpm; temperature, 23–25 °C; humidity, 25–35%. After fabrication, 3D matrices were removed from the collector, dried in vacuum under 10 Pa for 12 h, and stored in sealed zip-lock polyethylene containers at 4 °C.

### 2.3. Characterization of Matrices

#### 2.3.1. Mechanical Testing of Matrices

Strain-stress diagrams were obtained using a universal Zwick/Roell Z100 (Zwick Roell, Ulm, Germany) test bench as described in ISO 7198:1998 [25]. Electrospun matrices were carefully cut into 10 mm × 50 mm rectangular shapes and placed between holders at a distance of 2–2.5 cm. Tensile strength testing was conducted at a rate of 10 mm × min^−1^ at room temperature (21–23 °C). At least four specimens of each sample were tested. The residual load after two-fold elongation of matrices was measured in the same way, with the difference that after matrix elongation, the load was removed, the clamps of the tearing machine were returned to their original position (l_0_), and the load was re-measured after two-fold elongation of the matrix.

#### 2.3.2. Study of 3D Matrix Surface Microstructure

The microstructure of the matrix surface was studied by scanning electron microscopy (SEM) as described earlier [26]. The fiber diameter and pore size were evaluated from the SEM images according to ISO 7198:1998 [25]. To assess the stability of the fiber structure, the 3D matrices were incubated in phosphate buffered saline (PBS) (Sigma-Aldrich, USA) or human plasma at room temperature for 27 days. After the incubation, the matrices were rinsed with H_2_O, air-dried, and examined by SEM.

#### 2.3.3. X-ray Photoelectron Spectroscopy

The X-ray photoelectron spectroscopy (XPS) study was performed on an SPECS electron spectrometer equipped with a PHOIBOS-150 MCD-9 hemispherical analyzer and a non-monochromatic twin Mg-Al source (SPECS GmbH, Berlin, Germany). To avoid thermal degradation of the sample, the X-ray gun was positioned at a distance of 30 mm from the sample holder and operated at an MgKα irradiation power of 70 W or less. The spectra were recorded with pass energy set to 50 eV (survey scans) and 10 eV (high-resolution ones). Before the measurements, the energy scale was calibrated using the Au4f_7/2_ (84.00 eV) and Cu2p_3/2_ (932.67 eV) peaks from gold and copper foils. The residual gas pressure during the spectra acquisition did not exceed 3 × 10^−7^ Pa. The samples were mounted on steel sample holders by using 3M^®^ copper conductive double-sided adhesive tape. Quantitative data processing was performed using the XPSPEAK software version 4.1 and atomic sensitivity factors reported previously [27]. To study the influence of medium on 3D matrices, they were incubated in water or ethanol for 48 h, as described in 2.4, rinsed, air-dried, and stored as described in 2.2.

#### 2.3.4. Additional Physicochemical Characteristics of Matrices

The contact angle was measured on a Drop Shape Analyzer–DS A25 (Kruss GmbH, Hamburg, Germany) using water as a solvent (drop volume, 1 µL; shooting speed, 160 frames per second). The porosity of the matrices was evaluated using matrix volume and PCL density according to the formula:

Porosity (%) = [1 − D_a_/D_p_] × 100, where D_a_ is the apparent density (matrix weight/matrix volume) and D_p_ is the polymer density.

SEM data was also used for porosity calculations as described in ISO 7198:1998 according to the formula:

Porosity (%) = [A_p_/(A_m_ + A_p_)] × 100, where A_p_ is the pore area and A_m_ is the matrix area.

Water absorption and weight loss of matrices were evaluated as described in ISO 7198:1998 according to the formulas:

Water absorption (%) = (W_w_ − W_o_)/W_d_ × 100;

Weight loss = (%) = (W_w_ − W_d_/W_o_) × 100, where W_w_ is the weight after wetting, W_o_ is the weight before wetting, and W_d_ is the weight after wetting and drying under vacuum. The accuracy of measurements considering 3D matrices weight and accuracy of weighting was about 1.5%. Weight loss was evaluated after 27 days of incubation of 3D matrices in PBS as described in Section 2.3.2.

The drying rate of the matrices was evaluated after soaking the matrices in water for 24 h, followed by weight measurements on a microbalance with an accuracy of ±0.1 mg.

### 2.4. Assessment of Paclitaxel Release

To evaluate the PTX release, 10 mm disks were excised from matrices by die cutting, weighed with an accuracy of ±0.1 mg, and placed in wells of a 48-well plate. Disks were covered with 250 µL of PBS or EDTA-stabilized human plasma (HBP). The plate was sealed with a sealed adhesive film (Microseal^®^ ‘B’ PCR Plate Sealing Film, adhesive, Bio-Rad, Hercules, CA, USA) to prevent drying, followed by incubation on a Titramax 1000 shaker (Heidolph, Schwabach, Germany) at 37 °C and platform rotation speed of 200 rpm for different times of up to 27 days. Two types of ^3^H-PTX release kinetics were evaluated. For Series 1, the matrices were incubated with the solution for 20 min, 60 min, 3 h, 9 h, 27 h, 3 days, 9 days, and 27 days without medium replacement. For Series 2, at each time point, the supernatant was removed, the matrix was rinsed, and incubated in a fresh replacement of the respective solution until the next time point, when the same procedure was repeated. After the incubation, the matrices were washed with distilled water and air-dried at room temperature. Radioactivity of the supernatants was measured in duplicate as described in Section 2.1. The concentration of PTX in the solution was calculated from the specific radioactivity of the preparation, assuming that one disc contained ~0.36 μg of PTX.

The influence of the matrix deformation on the ^3^H-PTX release was studied as follows. A strip of a 3D matrix was fixed in clamps, the distance between the clamps was measured, and subsequently matrix was slowly stretched with a screw to double the measured distance. After removing the load, linear sizes of the strip were measured in order to recalculate amount of PTX per square cm, discs were excised from the deformed matrix, and ^3^H-PTX release was evaluated as described. All experiments were performed in duplicate.

### 2.5. Statistical Processing of Data

Microsoft Excel 2010 was used to handle and process the experimental data. Statistical analyses were performed using the Statistica 7.0 package (StatSoft Inc., Tulsa, OK, USA).

## 3. Results and Discussion

### 3.1. Synthesis of Radioactively Labeled PTX

^3^H-PTX was synthesized by the thermoactivated tritium exchange as described earlier [24]. The RP-HPLC-purified ^3^H-PTX preparation was obtained with a specific radioactivity of 1.5 mCi/mL (~0.3 Ci/mM of PTX). According to the TLC data, the compound was homogenous and detected as one spot on the autoradiograph with Rf as non-labeled PTX. Preliminary tests were performed to determine the optimal conditions for the measurement of the sample radioactivity in PBS and HBP. It was shown that the counting efficiency is inversely proportional to the volume of the sample; after the addition of the sample to scintillator at a ratio of 1:10 (*v*:*v*),the no turbidity of the solution was detected, along with maximal efficacy of tritium detection independently of sample origin (H^3^ was detected with practically equal efficacy both in PBS and in human plasma). Therefore, the radioactivity of all samples was evaluated in the same way, i.e., 0.1 mL of the sample was diluted in the scintillator up to a total volume of 1 mL. The molar concentration of PTX was evaluated using the total specific radioactivity of the (^3^H-PTX + PTX) preparation introduced into the matrix, which was ~47,000 cpm/nM.

### 3.2. Electrospinning and Characterization of 3D Matrices Prepared from Different Mixtures of PCL with HSA and Solvents

HSA is known to be the main protein in the blood plasma, which binds PTX into two types of complexes with a common binding constant, K = 1.43 × 10^4^ M^−1^ [21], and carries it in the blood. In electrospun 3D matrices prepared from PCL-HSA mixtures, a significant amount of matrix-bound HSA is located on the fiber surface and remains exposed for a long time [26]. We consider that such matrices could be used as a coating for bare-metal vascular stents and delivery of PTX into the vascular wall.

Conditions for preparation of matrices from PCL with HSA, PTX, and DMSO by electrospinning are presented in Table 1.

The strength of the PCL-based matrices was 3–5.1 MPa depending on the composition of the electrospinning solution. The regions of elastic and plastic deformation for PCL-HSA matrices (including those produced from HFIP with DMSO) were 8 ± 1.5% and 290 ± 16%, respectively, which indicated their strength and elasticity compared to matrices prepared without the protein. Residual load after two-fold elongation was 1.4 ± 0.16 to 0.35 ± 0.07 MPa in the dry matrices and 1.0 ± 0.11 to 0.26 ± 0.24 MPa in the wet matrices prepared from PCL/PTX/10% HSA and PCL/PTX/10% HSA/6% DMSO, respectively. The strength of the coronary artery wall is 1–2 MPa depending on the age of the donor and the presence of atherosclerotic lesions [28]. The thickness of the coronary artery wall is at least 2 mm [29], and the thickness of the stent coating is 0.1–0.15 mm. Therefore, the residual load in the coating of the stent after its installation does not exceed 5% of the load provided by the vascular wall. If the coatings for the metal stents are prepared from the solutions of PCL with HSA and DMSO, the load applied by the matrix on the stent struts will be no more than 1.5–2% of the load from the wall of the stented artery. Hence, from a mechanical point of view, the applicability of PCL-HSA 3D matrices for the covering of vascular grafts was demonstrated.

**Figure 1:**
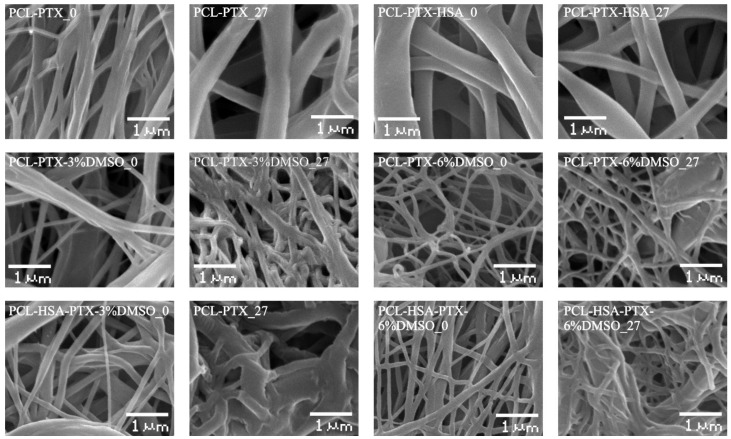
Microstructure of 3D matrices (SEM, ×3000 magnification). The digit following the matrix composition indicates the incubation time of the matrices in PBS.

SEM analysis revealed that all prepared matrices were composed of microfibers with diameters determined by the composition of the electrospinning solution (Figure 1). Matrices from PCL and PCL with HSA consisted of fibers with average diameters of 0.32 and 0.56 µm, respectively (Table 2). The addition of DMSO decreased the average fiber diameter (Figure 1, Table 2). The diameter of the fibers in 5% PCL/PTX/3% DMSO/HSA matrices was 0.37 ± 0.08 µm, which was the highest of all matrices with DMSO (from 0.13 to 0.19 µm). The fibers of all matrices had a smooth surface. Using × 40,000 magnification, with which structures as small as 20 nm can be resolved, no pores of a diameter of 10–20 nm could be detected on the fiber surface, although a slight roughness probably caused by the drying of the solvent was observed.

The porosity of the fiber surface and the dependence of the pore size on electrospinning conditions, such as a solvent, polymer, and wetting, were studied previously [30,31]. It was shown that pore formation is caused by the evaporation of the solvent and cooling of the surface of a newly formed fiber (thermally induced phase separation, TIPS) accompanyied by condensation of water vapor (water induced phase separation, WIPS). The occurrence of pores only on the fiber surface as demonstrated previously by TEM and atomic force microscopy (AFM) is evidence in favor of WIPS rather than TIPS as the main determinant of fiber porosity [32]. Obviously, the interplay of solvent diffusion in polymers, polymer solubility in solvents, and phase transitions occurring for several polymers in the same solvent can play an important role in the formation of the structure and the surface of the fibers. Pore formation can lead to a significant increase in the surface area of electrospun 3D matrices of up to ~100–1000 m^2^/g [31], which undoubtedly will affect the rate and extent of drug release.

Notably, it is often postulated that the nanopores in fibers are tortuous rather than straight and have a significant length [33]. Their size depends, among other factors, on the type of solvent as well as the protein concentration and solubility. It should be noted that in the solvent systems, such as dimethylformamide/dichloromethane or dimethylformamide/chloroform [34], proteins are poorly dissolved and are present in suspension. This fact can significantly influence the fiber structure, exposition of the proteins on the fiber surface, and the release of the protein from the fiber. In contrast, proteins, including has, are readily dissolved in HFIP with the preservation of their 3D structure [26], and thus the structure of fibers produced form HFIP should significantly differ from those previously mentioned.

According to SEM, the incubation of matrices in PBS for 27 days had little effect on the structure of matrices made of pure PCL, PCL with HSA, and PCL with 6% DMSO. These results are consistent with the data obtained earlier, which showed that PCL matrices are stable for 12–24 months [35].

Shrinkage of the material with a thickening of fibers and increasing the number of interfiber contacts were observed in the matrices produced from PCL with 3% or 6% DMSO and with or without HSA. SEM demonstrates that the matrices with HSA tend to change morphology more than those without the protein (Figure 1). Apparently, the hydration of the fibers, which involves the hydration of the integrated has, is the main factor. In other words, HSA molecules within the fiber promote diffusion of the water, hydration, and swelling. It was demonstrated in our previous work that the size of the HSA molecule in water exceeds its size in HFIP, by applying small-angle X-ray scattering [26]. Since incubation does not change the weight of the matrices, no significant degradation of the matrices can be observed. However, the change and reorganization of the fiber structure and HSA hydration can have a considerable effect on PTX release.

**Figure 2:**
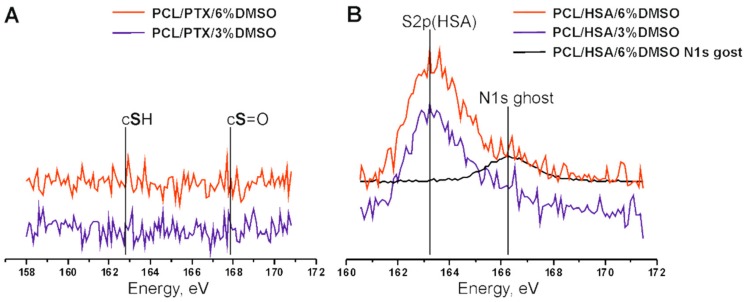
XPS spectra of the samples PCL/PTX/DMSO (**A**) and PCL/HSA/DMSO (**B**).

The concentration distribution of added components on the matrix surface was evaluated by XPS (Figure 2). To evaluate the residual amounts of DMSO S2p, XPS spectra of sulfur were acquired (Figure 2A). The use of S2p signals for spectroscopic analysis of PCL-HSA matrices is rather complicated, since sulfur is a minor component of proteins. Fortunately, “organic” (C-S-H) and sulfoxide (C-S=O) states of sulfur have substantially different S2p positions on a binding energy scale. Indeed, the spectra of PCL-HSA samples have a distinct S2p peak at 163.3 eV attributed to protein sulfur due to both its binding energy value and the signal intensity. At first glance, the smaller peak located at 166.3 eV could account for sulfur in DMSO moieties. However, it definitely includes N1s a “ghost peak”, resulting from cross-contamination by AlKα radiation (ca. 1% in this case). The intensity of N1s ghost line (black curve in Figure 2B) well matched the intensity of the peak of 163.3 eV in the S2p spectrum. Thus, the samples carried no sulfur-containing moieties on their surface regardless of DMSO content and treatment regimes, suggesting that PTX release could not be caused by the diffusion of DMSO from the matrices.

Table 3 shows that the HSA content of the surface layer slightly decreased inversely proportionally to the DMSO concentration, which may indicate that HSA is transported by the HFIP flow during fiber drying. As shown earlier [26], incubation of matrices in physiological solution leads to a reorganization of the surface layer and an increase in the HSA concentration on the surface.

According to XPS results, the PTX concentration in the surface layer of the PCL-based 3D matrices also decreased inversely proportionally to DMSO concentration in ES solution (Table 2). Only 3.7% of PTX was detected in the surface layer of 5%PCL/PTX/6%DMSO 3D matrices in contrast to 21% in the matrices from pure PCL. The increase in the PTX concentration on the surface of electrospun fibers was previously described for matrices prepared from PCL in dichloromethane [12]. The authors attributed this finding to the effect of solvent drying during ES.

Similarly to HSA, PTX concentration in the surface layer increased after incubation of the matrices in the physiological solution, which was especially noticeable in the 5% PCL/ PTX/6% DMSO matrices made (10% PTX concentration increase). Apparently, wetting of the matrices leads to a structural reorganization of the fiber surface, and low PTX solubility in water [35] results in its accumulation on the matrix surface. Unfortunately, one is unable to use XPS to measure concentrations of PTX and HSA simultaneously. Considering the ability of HSA to bind PTX (in aqueous solution) and potential hydrophobic interactions between PTX and HSA one can consider that HSA exposed at the fiber surface can additionally retain PTX, slowing down its release out of fibers.

The detection depth of XPS is no more than 10 nm. In a 500 nm fiber, the 10 nm thick surface layer occupies approximately 8% of the total volume. Given that the percentage of PTX in the matrices is no more than 0.01%, even if all PTX were to concentrate in this layer, its concentration should not exceed 0.2%, as opposed to the 3.7–21% PTX concentration according to XPS data. For the weight balance to equalize, the PTX layer should have a depth of 1 nm (almost monolayer). Thus, a significant part of PTX is concentrated in the thin surface layer of the fibers, which interfaces with the external medium and facilitates PTX release into the solution.

The porosity of matrices calculated from the matrix volume and the density of the polymer composition varied in the range 77–80% and was not significantly different for matrices prepared from different solutions. The porosity evaluated by SEM was 54% to 76%, and the pore size varied from 5.7 to 0.97 µm. The matrices synthesized from DMSO solutions were less uniform, in terms of not only fiber diameter, but also the pore size (Table 2, Figure 1).

No weight loss of the matrices was detected after drying, even though matrices made of PCL with 10% HSA are known to release the protein into solution [26]. This loss could not be detected by gravimetric methods under the conditions of the experiment (the loss is 0.2–0.3% of the matrix weight, provided that the matrix weight is no more than 5 mg, and the weight of released HSA is only 10–15 µg).

Water absorption varied from 294% for the pure PCL matrices to 883% for 5% PCL/PTX/6%DMSO/HSA matrices. According to a previous study based on the Washburn’s equation, the time required to fill nanopores is several microseconds [36], which means that the observed differences in water absorption cannot be caused by incomplete hydration of the matrices. Obviously, water absorption depends not only on the contact angle and fiber diameter, but is also dictated by the nanostructure of the fibers. In addition, HSA-containing matrices retain more water than those without the protein, which is likely caused by the higher hydrophilicity of the HSA-containing matrices and the hydration of HSA within the fibers.

**Figure 3:**
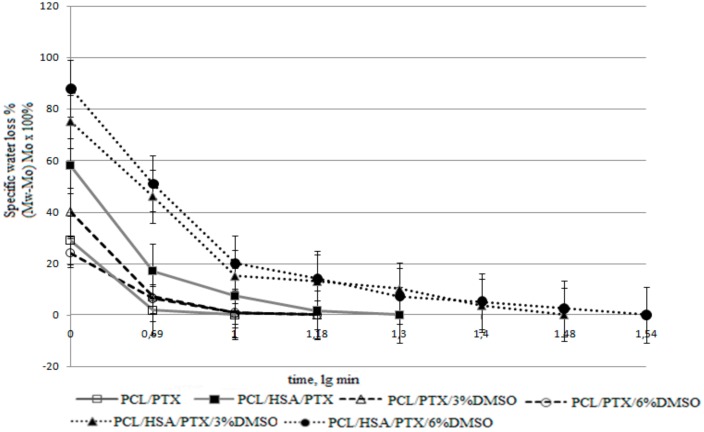
Drying rate of 3D matrices. The data presented as means, error of the mean does not exceed 7%.

The surface of PCL-based matrices without HSA was hydrophobic (Table 2), they were poorly wetted, and lost almost all water after the first drying period (Figure 3) [36]. The second drying period for these matrices was very short, which can indicate a rapid transport of water from the internal volume of the matrices and lack of interaction between the water and matrix surface.

PCL/HSA matrices had a contact angle below 90°, indicating hydrophilicity. Provided that the density of PCL is 1.021 g/cm^3^, the matrix adsorbed no less than 589% of water (weight percentage), but quickly lost it during the first drying period. The hydrophilic fiber surface retains the water to a greater extent, therefore, explaining the longer second drying period typical for these matrices.

PCL/HSA/DMSO matrices were more hydrophilic compared to those without HSA and absorbed significantly more water (Table 2). Apparently, the mass transfer of water in these matrices is determined not only by the flow from the hydrostatic drop pressure occurring due to filling of pores of different radii (as described by Laplace’s law), but also the interaction of water with the fiber surface. Indeed, mass transfer may include surface-diffusion flows, film formation, and the occurrence of disjoining pressure, which can lead to fluid transfer in surface films when their thickness deviates from the equilibrium value (surfaces with ultrastructural disturbances are filled preferentially) [36]. Matrices with HSA and DMSO exhibited pronounced first and second drying periods, with the latter lasting longer and resulting in a higher water loss as compared to matrices without DMSO (Figure 3). Judging by this, the microstructure of such matrices has similar characteristics, although matrices with 6% DMSO had a lower contact angle and were more thoroughly filled with water. These matrices were also more heterogeneous in fiber diameter (Figure 1), consisted of thinner fibers, and had a more branched surface.

### 3.3. PTX Release from Matrices

It is known that hydrophilic matrices have a lower capacity to adsorb proteins [37] and induce inflammation and, therefore, have advantages over hydrophobic matrices as materials for implants [38]. Introduction of HSA decreases the contact angle, thus making matrices more hydrophilic (Table 2), and increasing their hemo- and biocompatibility [22,23]. The introduction of 3% or 6% of DMSO in PCL matrices did not lead to a significant change in the contact angle, but affected matrix structure, fiber diameter, and water absorption, thus effectively increasing the area of medium-matrix interface. The two-phase nature of drying and high interface surface characteristic of the matrices with HSA and DMSO compared to other 3D matrices allow one to hope that PTX can be released effectively from such matrices and that its release kinetics would also follow a two-phase profile.

As mentioned in the “Materials and Methods”, PTX release from the matrices was studied without (1) or with the replacement of the media (2), i.e., the solution was replaced by the new portion after each time point. The selected conditions emulate PTX release into the bloodstream (medium replacement) and in the vascular wall (no or slow replacement of medium).

It should be noted that mathematical modelling of the mechanisms of drug delivery controlled by diffusion, osmosis, swelling/dissolution processes, etc. is well-developed [39]. The release of low-molecular-weight compounds (Rhodamine 610) from electrospun matrices made of PCL [33] or bovine serum albumin (BSA) from PCL/BSA matrices [40] was studied earlier. The delivery of rhodamine was shown to be limited by its diffusion from fibers. The authors developed a theoretical model of the release and evaluated its parameters, such as nanoporosity and desorption enthalpy. They showed that the release is saturable-only a portion of rhodamine can be released, the precise amount depending on the fiber pore structure. Diffusion of this part of the compound from the fibers took between two and four days, and the fibers no longer release rhodamine. Materials exhibiting this type of the release are poor candidates for stent coatings. Moreover, the authors did not attempt to enhance the bio- and hemocompatibity of the matrices and only studied the release of rhodamine and protein molecules from synthetic polymers.

**Figure 4:**
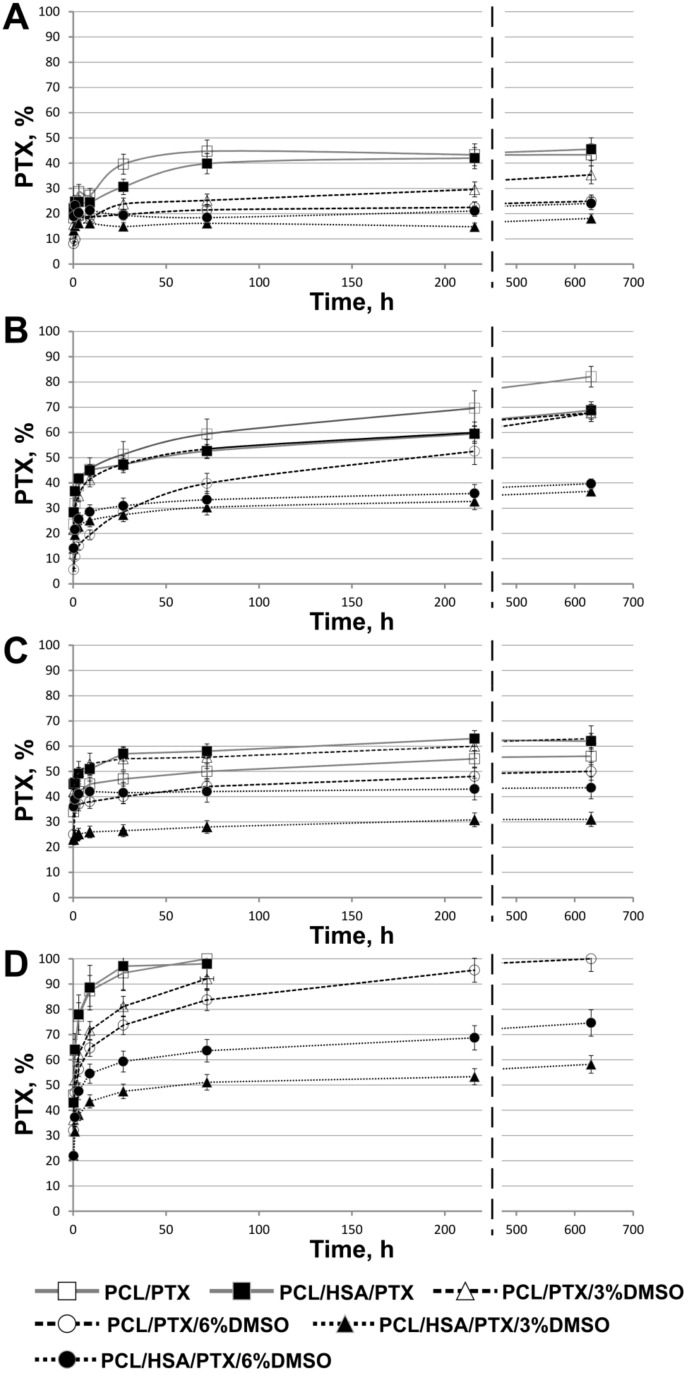
Kinetics of PTX release from matrices. (**A**) Incubation of 3D matrices with PBS without medium replacement; (**B**) incubation of 3D matrices with PBS with medium replacement after each time point; (**C**) incubation of 3D matrices with human plasma without medium replacement; (**D**) incubation of 3D matrices with human plasma with medium replacement after each time point.

The kinetic curves of PTX release are presented in Figure 4. The release in PBS from 5%PCL/PTX and 5% PCL/PTX/10%HSA matrices appears to be similar to that described earlier [33] with a saturation time of three days (Figure 4A). However, some differences were observed, which were probably caused by the low solubility of PTX (less than 0.3 mg/L or 3.5 µM in water). When incubated with medium replacement, PTX was released from the fibers past the third day and for as long as up to 27 days, and the total amount released was double the PTX release in PBS without medium replacement (Figure 4B).

In a fiber, PTX can be found both in the volume and on the surface: PTX_total_ = PTX_volume_ + PTX_surface_. One can assume that without medium replacement, an equilibrium between PTX on the surface and PTX in the solution is achieved during the first three days (in PBS). Because of the low PTX solubility in water, the rate at which the equilibrium is established is limited by its desorption from the matrix surface. In the case of medium replacement, dissolved PTX is periodically removed, leading to additional desorption of PTX from the fibers into the solution, thus increasing the amount of dissolved PTX over time. Moreover, in HSA-containing matrices, the sorption/desorption on the fiber surface is accompanied by the binding of PTX to HSA (Kа = 1.43 × 10^4^ M^−1^) [21], which can retain PTX both within and on the surface of the fibers.

During the incubation of matrices with plasma without replacement, the nature of the release did not change, but saturation was achieved in five to seven days (Figure 4C) and more PTX was released (55–60% vs. 45%). When incubated with plasma replacement, PTX was completely released from matrices in three days (Figure 4D). Apparently, HSA in plasma binds dissolved PTX, which prevents the resorption of PTX on the matrix surface. The concentration of HSA-bound PTX can be evaluated by considering the following factors: HSA concentration in plasma is ~1 mM; PTX concentration is ~0.82 µM; when 50% of this compound is released from the matrix (MW_PTX_ is 854, the matrix contains 0.36 µg, the solution volume is 0.25 mL); and the association constant is Kа = 1.43 × 10^4^ M^−1^. Using material balance equations, the concentration of the PTX-HSA complex is calculated as 0.77 µM. Thus, under these conditions, 94% of released PTX is associated with HSA. It is likely that other biomolecules in plasma can also be involved, e.g., lipids can interact with fat-soluble PTX on the surface or even within the matrix.

Since incubation with plasma replacement increased the PTX release to 100%, the PTX diffusion rate within the fibers/matrix did not limit the rate of PTX release. It should be noted that, according to SEM data, the fiber structure of these 3D matrices also did not change during long term incubation and PTX release into the solution (Figure 1); that is, the degradation or reorganization of the matrix structure is not associated with long-term PTX release.

The results indicate that 5%PCL/PTX and 5%PCL/PTX/10%HSA matrices are not suitable for the prolonged delivery of the drug because they do not facilitate the long time two-phase kinetics of PTX release, and the drug is rapidly released when the matrix is in contact with human plasma. To obtain 3D matrices, which are capable of prolonged PTX release, it is necessary to either change the fiber structure or alter the PTX distribution in the fibers. To retain PTX in the fiber during the solvent drying step and/or obtain the porous fibers, DMSO was added to the electrospining solution because it is nontoxic, has a high boiling point (189 °С), and dissolves PTX. In previous reports, the factor of matrix nanoporosity has been theoretically explored [31]. The authors of this report used a similar solvent system for electrospinning—a mixture of dimethylformamide (T_bp_ = 153 °С) with dichloromethane (T_bp_ = 40 °С). It was shown that the addition of 3% DMSO in the electrospinning solution significantly decreased the total PTX release into PBS (up to 25–35%) under conditions without medium replacement (Figure 4A). For matrices with 6% DMSO, the PTX release was slightly decreased, while almost no decrease of the total PTX release was observed in medium replacement conditions (Figure 4B). These data are consistent with XPS demonstrating a decrease of PTX on the surface of 3D matrices electrospun from the solutions with DMSO (Table 3). As in previous cases, the rate of PTX diffusion from the fiber/matrix volume to its surface does not limit the rate of PTX transfer into the solution since no change in the total release of PTX under the conditions with medium replacement was detected.

In regards to PTX release into the plasma, the addition of 6% of DMSO slightly decreased the release under the conditions with plasma replacement (Figure 4D), but had no effect on PTX release without replacement (Figure 4C). It should be noted that the matrices prepared from DMSO-containing solutions consist of thinner fibers as compared with the other matrices. Moreover, 3D matrices produced from ES solutions with 6% of DMSO are characterized by more hydrophobic surfaces, lower water adsorption, and lower amount of PTX on the surface according to the XPS data as compared with the matrices prepared from the solutions containing 3% DMSO.

Generally speaking the matrices prepared from solutions containing DMSO and HSA are characterized by a slow release of PTX (Figure 4C,D). Matrices made from the 5%PCL/PTX/3%DMSO/10%HSA released ~30% and 60% of PTX in 28 days under the conditions, both with and without medium replacement, respectively. The daily release of PTX decreased from ~25% (day 1) to 0.05% (day 28) and from ~45% (day 1) to 0.26% (day 28) under the conditions, both with and without medium replacement, respectively. These results indicate the effect of DMSO, which changes the matrix structure and the distributions of both PTX and HSA in the fibers (Table 2 and Table 3).

Thus, the composition of ES solution and outside medium significantly affects the rate of the PTX release. The removal of dissolved PTX due to the medium replacement and/or binding to HSA both prevent its resorption and lead to the fast diffusion of PTX from the fibers and its complete release from the matrix. Thus, desorption of PTX from fibers is the rate-limiting stage of PTX release. The results for matrices produced from ES solution with DMSO show that increased hydrophobicity of 3D matrices reduces desorption of PTX from the fiber surface. In addition, HSA in PCL fibers has a more pronounced PTX retention effect. 3D matrices produced from solutions of PCL, HSA, and DMSO in HFIP exhibited a pronounced two-phase kinetics profile of PTX release into PBS or human plasma. The first phase is the fast release due to desorption of PTX from the surface, while the second stage is the slow PTX release caused by its interaction with HSA located within the hydrophobic fibers.

Earlier, it was shown that rhodamine was only partly released into aqueous solutions from electrospun fibers prepared from PCL solutions in dimethylformamide/dichloromethane mixture, and a portion of rhodamine localized in the solid polymer phase is never released [33]. Our results demonstrate that PTX can completely diffuse from the matrices prepared from PCL or PCL/HSA in HFIP with or without DMSO when the matrices are incubated with the human plasma (Figure 4D). According to the XPS data (Table 2), a significant amount of PTX is exposed on the matrix surface, and the incubation of matrices with aqueous solutions led to a release of PTX (Figure 4) and reorganization of the surface layer, resulting in a further increase in the PTX concentration on the matrix surface (Table 2). Apparently, the structure of the fibers prepared from HFIP solutions and chemical properties of PTX allow this compound to be redistributed in the fibers. In any case, the matrices capable of complete PTX release are optimal for facilitating its delivery.

The data on the structure of 3D matrices (Table 2 and Table 3) are strongly correlated with the release of PTX from the fibers. Actually, fibers able to accumulate PTX in their bulk and swelling during incubation demonstrated slower PTX release kinetics. It is interesting to note that water loss by matrices correlated with the kinetics of PTX release (Figure 3 and Figure 4).

Stretching of the matrices to the point of two-fold elongation did not affect the PTX release. Both the nature of the release and the amount of released PTX per one unit of the matrix weight remained unchanged. However, the elongation of the matrices led to plastic deformation because of the small area of elastic deformation (7–10%), and the linear size of the matrices increased to 125–185% after loading. The amount of PTX released from elongated matrices was proportionally lower compared to untreated matrices. It is necessary to take this into account when optimizing the cytostatic dose of the drug for stent coating.

The nature of PTX release from 3D matrices containing HSA and DMSO and electrospun from HFIP solutions enables their use as coatings for vascular stents intended to prevent restenosis and proliferation of cell in the vascular wall. The perivascular delivery of PTX at concentrations from 20 to 230 µM under the conditions of internal pressure ensures its efficient accumulation in the artery walls, with predominant localization in the adventitia area [41]. According to some authors, the diffusion coefficient for PTX in the arterial wall varies from 1 × 10^−8^ cm^2^/s to 4.87 × 10^−6^ cm^2^/s [42]. It is interesting to note that the diffusion coefficient for HSA (the main transporter of PTX) in the aortal wall (evaluated without regard to potential binding in the adventitia) is 1.06 × 10^−8^ cm^2^/s [43] and the protein is retained in the region of elastin fibers localized in the smooth muscle layer of the adventitia [44]. In addition, it has been shown that elastin itself binds PTX and can promote its retention in the artery wall [45]. The accumulation of PTX in this layer can be mediated by PTX binding to both HSA and elastin. The toxic PTX concentration against smooth muscle myocytes is ~10 nM [46]. Providing that the matrix contains 0.46 µg/cm^2^ of PTX and 1% of it is released daily, the PTX concentration in the wall will be equal to or higher than its toxic concentration. One should consider the initial accumulation of PTX in the wall, its low solubility, and binding with the components of the extracellular matrix. Thus, the coating of bare-metal stents with scaffolds electrospun from the solutions of PCL with HSA and DMSO and containing 0.46 µg/cm^2^ of PTX can be toxic against vascular wall myocytes for at least three months. Herewith, the PTX dose, which is released during the first days after the stent implantation, will make it possible to eliminate the proliferation of activated cells, and a high PTX concentration will compensate for its diffusion through the partially destroyed artery wall immediately after implantation. Furthermore, the data on the complete PTX release from the matrices produced from the HFIP solutions of PCL allows one to hope for an even more prolonged release of PTX from the fibers. It should also be noted that the change in the structure of the matrices during incubation, their shrinkage, and fiber aggregation, resulting in the reduction of the phase interface, could prolong the PTX release from the fibers and thus assist the cytotoxic effect of drugs introduced in such matrices.

## 4. Conclusions

The physicochemical properties of electrospun matrices prepared from the solutions of PCL with PTX in HFIP and their blends with HSA and DMSO were studied. It was shown that 3D matrices produced from a HFIP solution of PCL with PTX, HSA, and DMSO are the most suitable to be used as coatings for bare-metal stents because they are not expected to exert any significant additional stress on the stent beams, exhibit long time two-phase kinetics of PTX release, and thus are expected to be able to maintain a cytotoxic PTX concentration in the vascular wall for at least three months. It was shown that PTX can be completely released from these matrices without fiber degradation. The use of plasma as the external medium accelerated PTX release, while two-fold elongation of 3D matrices did not interfere with the kinetics of the release. Thus, PCL-based 3D matrices containing HSA, PTX, and DMSO can be used to produce coated vascular stents with prolonged delivery of PTX.

## Figures and Tables

**Table 1:** Electrospinning conditions for fabrication of 3D matrices.

Matrix Composition	Electrospinning Parameters		
	Voltage, kV	Feed Rate of the Solution, mL/h	Distance between Electrodes, cm
PCL/PTX	23.0	1.2	20
PCL/PTX/10% HSA	23.5	1.3	20
PCL/PTX/3% DMSO	23.0	1.3	20
PCL/PTX/6% DMSO	24.5	1.3	20
PCL/PTX/10% HSA/3%DMSO	24.5	1.4	20
PCL/PTX/10% HSA/6% DMSO	25.0	1.4	20

**Table 2:** Physico-chemical properties of 3D matrices.

No	Sample	Fiber Diameter, µm	Pore Diameter, µm	Porosity, % *	Contact Angle, ° **	Water Absorption, %	Weight Loss, %
1	5% PCL/PTX	0.31 ± 0.04	5.72 ± 2.42	78/54.1	127.33 (±1.30)°	294 ± 7	0
2	5% PCL/PTX/10% HSA	0.56 ± 0.09	2.66 ± 1.21	77/61.4	88.89 (±3.03)°	589 ± 16	0
3	5% PCL/PTX/3% DMSO	0.19 ± 0.03	2.01 ± 0.73	78/65	128.30 (±2.18)°	400 ± 11	0
4	5% PCL/PTX/6% DMSO	0.13 ± 0.02	0.97 ± 0.32	80/76.6	132.35 (±3.11)°	238 ± 9	0
5	5% PCL/PTX/3% DMSO/HSA	0.37 ± 0.08	1.97 ± 0.52	77/71	124.73 (±3.49)°	750 ± 13	0
6	5% PCL/PTX/6% DMSO/HSA	0.16 ± 0.03	1.35 ± 0.40	79/61.3	120.52 (±2.66)°	883 ± 15	0

The data are presented as the mean ± error of the mean; * the first and second numbers are the porosity calculated from the apparent matrix density and the SEM data, respectively; ** the contact angle was evaluated as a mean of at least five measurements in different parts of the matrix.

**Table 3:** XPS data on HSA and PTX surface concentrations in electrospun 3D matrices obtained from different PCL/HSA or PCL/PTX blends.

No	Sample of 3D Matrix	Concentration of HSA or PTX, %	
		Initial Matrix	Matrix after Incubation in PBS
1	PCL/10% HSA	20 *	24 *
2	5%PCL/10%HSA/3%DMSO	18.9	27.1
3	5%PCL/10%HSA/6%DMSO	16.3	21.5
4	5%PCL/PTX	21.1	23.4
5	5%PCL/PTX/3%DMSO	15.1	23.9
6	5%PCL/PTX/6%DMSO	3.7	13.7

* From Ref. [26].
